# Design, synthesis and evaluation of novel cinnamic acid derivatives bearing *N*-benzyl pyridinium moiety as multifunctional cholinesterase inhibitors for Alzheimer’s disease

**DOI:** 10.1080/14756366.2016.1256883

**Published:** 2017-06-06

**Authors:** Jin-Shuai Lan, Jian-Wei Hou, Yun Liu, Yue Ding, Yong Zhang, Ling Li, Tong Zhang

**Affiliations:** a Experiment Center of Teaching & Learning, Shanghai University of Traditional Chinese Medicine, Shanghai, China;; b School of Pharmacy, Shanghai University of Traditional Chinese Medicine, Shanghai, China

**Keywords:** Alzheimer’s disease, β-amyloid aggregation, benzylpyridinium, cinnamic acid, metal chelator, neuroprotection

## Abstract

A novel family of cinnamic acid derivatives has been developed to be multifunctional cholinesterase inhibitors against AD by fusing *N*-benzyl pyridinium moiety and different substituted cinnamic acids. *In vitro* studies showed that most compounds were endowed with a noteworthy ability to inhibit cholinesterase, self-induced A*β* (1–42) aggregation, and to chelate metal ions. Especially, compound **5l** showed potent cholinesterase inhibitory activity (IC_50_, 12.1 nM for *ee*AChE, 8.6 nM for *h*AChE, 2.6 μM for *eq*BuChE and 4.4 μM for *h*BuChE) and the highest selectivity toward AChE over BuChE. It also showed good inhibition of A*β* (1–42) aggregation (64.7% at 20 μM) and good neuroprotection on PC12 cells against amyloid-induced cell toxicity. Finally, compound **5l** could penetrate the BBB, as forecasted by the PAMPA-BBB assay and proved in OF1 mice by *ex vivo* experiments. Overall, compound **5l** seems to be a promising lead compound for the treatment of Alzheimer’s diseases.

## Introduction

Alzheimer’s disease (AD) is a fatal neurodegenerative disorder that is clinically associated with cognitive impairment, language skill loss and dementia^1^. To date almost 48 million elderly people are affected by AD, and this number is estimated to show unparalleled growth and increase to spread 131.5 million by 2050^2^. Although the etiopathogenesis of AD is unclear, multiple factors such as amyloid-*β* (A*β*) deposits, low levels of acetylcholine (ACh), *τ*-protein aggregation, dyshomeostasis of biometals and oxidative stress, play a vital role in the pathogenesis of AD^3^. The current therapeutic options against AD are composed by one *N*-methyl-d-aspartate (NMDA) receptor antagonist and three acetylcholinesterase (AChE) inhibitors, namely memantine (NMDA), donepezil, rivastigmine and galantamine (AChE)^4^. Nevertheless, these marketed drugs modestly alleviate the symptoms but cannot cure brain damage or stop neuronal degeneration.

AChE and BuChE play a role in cholinergic signaling. According to the cholinergic hypothesis, the decrease in ACh levels results in memory loss and cognitive impairment, and the clinical restoration of cholinergic function is believed to alleviate AD symptoms^5^^,^^6^. Furthermore, studies have illustrated that AChE interacts with A*β* through the peripheral anionic site (PAS) promoting the formation of steady AChE-A*β* complexes, which are more toxic than single A*β* peptides^7^. Therefore, dual-site AChE inhibitors may be promising AD drug candidates^8^^,^^9^.

Among multiple factors, neurotoxic A*β* plaques in the brain are a key contributing factor in the pathology of AD. A*β* (1–40) and A*β* (1–42) are the key isoforms of A*β* peptides. A*β* (1–42) may aggregate more rapidly and show stronger neuron cytotoxicity than A*β* (1–40) does^10^^,^^11^. Preventing the formation and accumulation of A*β* is a probable therapeutic strategy for AD. The dyshomeostasis of metal ions such as Cu, Fe and Zn is commonly observed in many critical aspects of AD^12^. The Cu^2+^ present in the brain at an abnormally high concentration interacts with A*β*, leading to the accelerated formation of neurofibrillary tangles (NFTs) and generate reactive oxygen species (ROS), which further induce oxidative impairment in the brain^13^. Moreover, abnormally high levels of redox-active metal ions, such as Fe^2+^ and Cu^2+^, in the brain may lead to the formation of ROS^14^. Notably, compared with the normal levels of metal ions in brain, the subtraction of physiologically essential metals may lead to risk. Consequently, reducing the abnormally high concentration of metals in the brain by chelating the metals is an additional logical approach for AD treatment. Oxidative stress also plays a crucial role in the development of neurodegenerative disorders such as AD. It has been hypothesised that the antioxidant defence system cannot neutralize oxidative species in elderly people^15^. The oxidative stress theory of ageing also suggests that oxidative damage plays an important role in neuronal degeneration^16^. Therefore, drugs that can scavenge oxygen radicals may be used to prevent AD.

Due to the pathological complication of AD, the multi-target-directed ligand (MTDL) design strategy has been proposed, and a range of compounds have been developed to act on various targets^17–21^. In AD, the effectiveness of multifunctional molecules with two or more distinct pharmacological properties being properly incorporated is higher than that of single-targeted drugs^22^. Therefore, ChEIs with multiple effects, such as A*β* disaggregation, neuroprotection and oxidative load reduction may be a significant approach for AD management.

Cinnamic acid derivatives are naturally occurring compounds that possess various pharmacological properties for diverse neurological disorders^23^. Ferulic acid (FA) and curcumin (Cur) are representative bioactive compounds, and they can prevent A*β* fibril aggregation, inhibit A*β*-mediated toxicity, scavenge ROS and reduce inflammatory effects both *in vitro* and *in vivo*^24–29^. Consequently, cinnamic acid could serve as a beneficial fragment in the designed MTDL, such as tacrine-FA hybrids, FA-memoquin hybrids, FA-carbazole hybrids, donepezil-FA hybrids, cinnamic-*N*-benzylpiperidine hybrids^30–34^.

**Figure 1.**
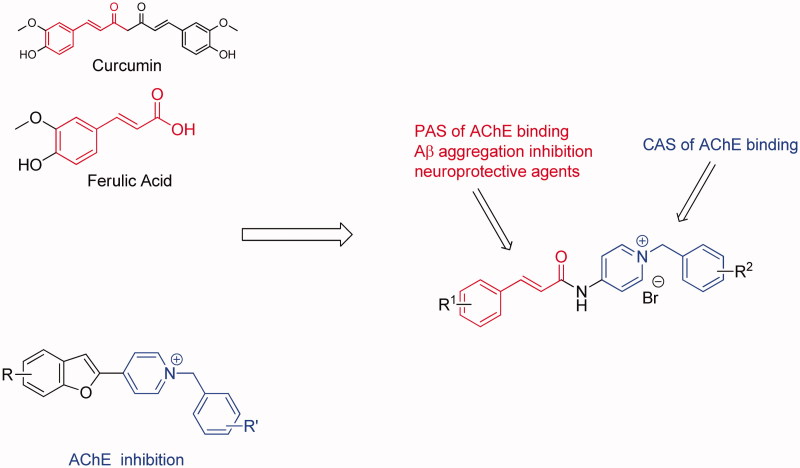
Drug design strategy for multi-target-directed ligands.

In the search for new cinnamic acid-based derivatives as anti-AD, we have focused on the structure of benzylpyridinium salts (Figure 1), which may represent a privileged scaffold that can be used to develop new AChE inhibitors^35–37^. Docking studies have shown that the *N*-benzyl pyridinium moiety interacts with the CAS of AChE, and the heterocyclic moiety interacts with the PAS of AChE forming stacking interactions. Proceeding with our researches on natural products with probable use as anti-AD^38–40^, we reasonably combined the cinnamic acid with benzyl-pyridinium to achieve new hybrids that are anticipated to be dual-acting AChE. In this study, all designed compounds were synthesised and evaluated for their biological activities, including their anti-aggregation activity towards A*β*, ChE inhibition, antioxidant activity, metal chelating properties, neuroprotective effects against A*β*-induced PC12 cell injury and the ability to cross the blood–brain barrier (BBB). Moreover, kinetic and molecular modelling studies were performed to further explore their mechanism of interaction with AChE and A*β*.

## Materials and methods

### Materials

All chemicals (reagent grade) used were purchased from Sino Pharm Chemical Reagent Co., Ltd. (Shanghai, China). Reaction progress was monitored using analytical thin layer chromatography (TLC) on precoated silica gel GF254 (Qingdao Haiyang Chemical Plant, Qing-Dao, China) plates and the spots were detected under UV light (254 nm). Melting point was measured on an XT-4 micromelting point instrument and uncorrected. IR (KBr-disc) spectra were recorded by Bruker Tensor 27 spectrometer (Billerica, MA).

^1^H NMR and 13C NMR spectra were measured on a BRUKER AVANCE III spectrometer (Billerica, MA) at 25 °C and referenced to TMS. Chemical shifts are reported in ppm (*δ*) using the residual solvent line as internal standard. Splitting patterns are designed as s, singlet; d, doublet; t, triplet; m, multiplet. The purity of all compounds was confirmed to be higher than 95% through analytical HPLC performed with Agilent 1200 HPLC System (Santa Clara, CA). Mass spectra were obtained on a MS Agilent 1100 Series LC/MSD Trap mass spectrometer (ESI-MS) (Santa Clara, CA).

#### General procedure for the preparation of compounds 3a–b

A mixture of compound **1** (10 mmol) and DMAP (10 mmol) in 20 mL anhydrous CH_2_Cl_2_ was stirred at 0 °C for about 10 min and EDCI (20 mmol) was added to the mixture and stirred at room temperature for 1 h. The compound **2** (10 mmol) was added to the solution, and stirring was continued overnight. The reaction mixture was diluted with H_2_O and extracted with CH_2_Cl_2_. The organic extracts were combined, washed with brine, and dried with anhydrous Na_2_SO_4_, and the solvent was evaporated *in vacuo* to give the crude product, which was purified by silica gel chromatography with CH_2_Cl_2_:MeOH =15:1 as an eluent to afford corresponding target compound as a yellow solid.

##### N-(pyridin-4-yl)cinnamamide (3a)

Cinnamic acid was reacted with 4-aminopyridine following the general procedure to give the desired product **3a** with a yield of 85%. ESI/MS *m/z*: 225.1 [M + H]^+^; ^1^H NMR (400 MHz, CDCl_3_) *δ* 8.75 (s, 1H), 8.50 (dd, *J* = 4.9, 1.4 Hz, 2H), 7.79 (d, *J* = 15.5 Hz, 1H), 7.64 (dd, *J* = 4.8, 1.6 Hz, 2H), 7.49 (dd, *J* = 6.6, 3.0 Hz, 2H), 7.40–7.32 (m, 3H), 6.63 (d, *J* = 15.5 Hz, 1H).

##### (E)-3-(3,4-dimethoxyphenyl)-N-(pyridin-4-yl) acrylamide (3b)

(*E*)-3*-*(3,4-dimethoxy -phenyl) acrylic acid was reacted with 4-aminopyridine following the general procedure to give the desired product **3b** with a yield of 88%. ESI/MS *m/z*: 285.2 [M + H]^+^; ^1^H NMR (400 MHz, CDCl_3_) *δ* 8.54 (d, *J* = 5.3 Hz, 2H), 7.96 (s, 1H), 7.75 (d, *J* = 15.4 Hz, 1H), 7.62 (d, *J* = 6.0 Hz, 2H), 7.15 (dd, *J* = 8.3, 1.8 Hz, 1H), 7.06 (d, *J* = 1.8 Hz, 1H), 6.90 (d, *J* = 8.3 Hz, 1H), 6.47 (d, *J* = 15.4 Hz, 1H), 3.95 (s, 3H), 3.89 (s, 3H).

#### General procedure for the preparation of compounds 5a–n

Compound **3** (10 mmol), appropriate benzyl chloride (12 mmol) and a catalytic amount of KI in dry acetonitrile (20 mL) was refluxed for 1–2 h. When the reaction was completed as indicated by TLC, the mixture was then concentrated under reduced pressure, and 20 mL of diethyl ether was added. On cooling, the precipitate was filtered and washed with diethyl ether to get the target compounds **5a**–**n** with high yields.

##### 1-Benzyl-4-cinnamamidopyridin-1-ium bromide (5a)

Yield 91%; yellow solid; IR (KBr) *ν* 3091, 3028, 2963, 1685, 1637, 1515, 1496, 1203, 1161, 966, 763, 752, 725 cm^−1^; m.p. >250 °C; ESI/MS *m/z*: 315.1 [M]^+^; ^1^H NMR (400 MHz, DMSO-d_6_) *δ* 11.90 (s, 1H), 8.98 (d, *J* = 6.7 Hz, 2H), 8.25 (d, *J* = 6.5 Hz, 2H), 7.79 (d, *J* = 15.7 Hz, 1H), 7.75–7.65 (m, 2H), 7.55–7.37 (m, 8H), 7.05 (d, *J* = 15.8 Hz, 1H), 5.74 (s, 2H). 13C NMR (100 MHz, DMSO-d_6_) *δ* 165.44, 152.24, 145.20, 145.20, 143.99, 134.68, 133.93, 130.81, 129.15, 129.15, 129.15, 129.15, 129.15, 129.15, 128.50, 128.50, 128.27, 128.27, 120.20, 115.21, 61.50.

##### 4-Cinnamamido-1-(3-methylbenzyl) pyridin-1-ium bromide (5b)

Yield 90%; yellow solid; IR (KBr) *ν* 3074, 3023, 2969, 1701, 1633, 1596, 1527, 1456, 1166, 965, 849, 743 cm^−1^; m.p. >250 °C; ESI/MS *m/z*: 329.1 [M]^+^; ^1^H NMR (400 MHz, DMSO-d_6_) *δ* 11.80 (s, 1H), 8.94 (d, *J* = 6.7 Hz, 2H), 8.22 (d, *J* = 6.4 Hz, 2H), 7.80 (d, *J* = 15.6 Hz, 1H), 7.70 (d, *J* = 4.9 Hz, 2H), 7.49 (d, *J* = 5.1 Hz, 3H), 7.33 (dd, *J* = 15.5, 8.0 Hz, 2H), 7.28 (d, *J* = 7.6 Hz, 1H), 7.24 (d, *J* = 7.5 Hz, 1H), 6.99 (d, *J* = 15.7 Hz, 1H), 5.67 (s, 2H), 2.32 (s, 3H). 13C NMR (100 MHz, DMSO-d_6_) *δ* 165.92, 152.72, 145.68, 145.68, 144.56, 139.03, 135.06, 134.42, 131.33, 131.03, 129.66, 129.66, 129.58, 129.51, 128.78, 128.78, 126.08, 120.66, 115.77, 115.77, 62.09, 21.39.

##### 4-Cinnamamido-1-(4-methylbenzyl) pyridin-1-ium bromide (5c)

Yield 88%; yellow solid; IR (KBr) *ν* 3015, 1697, 1628, 1509, 1458, 1339, 1162, 975, 860, 761 cm^−1^; m.p. >250 °C; ESI/MS *m/z*: 329.1 [M]^+^; ^1^H NMR (400 MHz, DMSO-d_6_) *δ* 11.84 (s, 1H), 8.93 (d, *J* = 6.9 Hz, 2H), 8.23 (d, *J* = 6.6 Hz, 2H), 7.79 (d, *J* = 15.7 Hz, 1H), 7.70 (d, *J* = 4.9 Hz, 2H), 7.49 (d, *J* = 5.1 Hz, 3H), 7.40 (d, *J* = 7.7 Hz, 2H), 7.26 (d, *J* = 7.6 Hz, 2H), 7.01 (d, *J* = 15.9 Hz, 1H), 5.67 (s, 2H), 2.31 (s, 3H). 13C NMR (100 MHz, DMSO-d_6_) *δ* 165.91, 152.68, 145.58, 145.58, 144.52, 139.18, 134.43, 132.16, 131.32, 130.18, 130.18, 129.65, 129.65, 129.08, 129.08, 128.77, 128.77, 120.68, 115.72, 115.72, 61.90, 21.22.

##### 4-Cinnamamido-1-(3-fluorobenzyl) pyridin-1-ium bromide (5d)

Yield 89%; white solid; IR (KBr) *ν* 3075, 3018, 2966, 1701, 1632, 1589, 1526, 1448, 1146, 965, 848, 750, 676 cm^−1^; m.p. >250 °C; ESI/MS *m/z*: 333.2 [M]^+^; ^1^H NMR (400 MHz, DMSO-d_6_) *δ* 11.84 (s, 1H), 8.96 (d, *J* = 6.7 Hz, 2H), 8.23 (d, *J* = 6.5 Hz, 2H), 7.80 (d, *J* = 15.8 Hz, 1H), 7.70 (d, *J* = 4.9 Hz, 2H), 7.54–7.45 (m, 4H), 7.42 (d, *J* = 9.7 Hz, 1H), 7.34 (d, *J* = 7.6 Hz, 1H), 7.28 (t, *J* = 8.6 Hz, 1H), 7.00 (d, *J* = 15.8 Hz, 1H), 5.74 (s, 2H). 13C NMR (100 MHz, DMSO-d_6_) *δ* 165.93, 163.72 (d, 1*J*_CF_=246.44 Hz), 152.85, 145.78, 145.78, 144.60, 137.61 (d, 3*J*_CF_=7.58 Hz), 134.41, 131.80 (d, 3*J*_CF_=8.29 Hz), 131.34, 129.66, 129.66, 128.79, 128.79, 120.64, 116.51 (d, 2*J*_CF_=21.72 Hz), 116.07 (d, 2*J*_CF_=22.15 Hz), 115.80, 61.27.

##### 4-Cinnamamido-1-(4-fluorobenzyl) pyridin-1-ium bromide (5e)

Yield 95%; yellow solid; IR (KBr) *ν* 3083, 3022, 2969, 1626, 1509, 1462, 1338, 1158, 964, 846, 767 cm^−1^; m.p. >250 °C; ESI/MS *m/z*: 333.1 [M]^+^; ^1^H NMR (400 MHz, DMSO-d_6_) *δ* 11.84 (s, 1H), 8.95 (d, *J* = 6.7 Hz, 2H), 8.23 (d, *J* = 6.5 Hz, 2H), 7.79 (d, *J* = 15.8 Hz, 1H), 7.70 (d, *J* = 5.2 Hz, 2H), 7.63–7.57 (m, 2H), 7.49 (d, *J* = 5.1 Hz, 3H), 7.31 (t, *J* = 8.4 Hz, 2H), 7.00 (d, *J* = 15.8 Hz, 1H), 5.71 (s, 2H). 13C NMR (100 MHz, DMSO-d_6_) *δ* 165.92, 163.15 (d, 1*J*_CF_=246.44 Hz), 152.76, 145.61, 144.56, 137.03, 134.42, 131.62 (d, 3*J*_CF_=7.98 Hz), 131.33, 129.65, 128.78, 120.66, 116.52 (d, 2*J*_CF_=21.37 Hz), 115.77, 61.19.

##### 1-(3-Bromobenzyl)-4-cinnamamidopyridin-1-ium bromide (5f)

Yield 92%; yellow solid; IR (KBr) *ν* 3087, 3020, 2966, 1708, 1634, 1595, 1516, 1165, 1165, 1136, 966, 837 761, 703 cm^−1^; m.p. >250 °C; ESI/MS *m/z*: 393.0, 395.0 [M]^+^; ^1^H NMR (400 MHz, DMSO-d_6_) *δ* 11.82 (s, 1H), 8.95 (d, *J* = 6.8 Hz, 2H), 8.23 (d, *J* = 6.5 Hz, 2H), 7.86–7.75 (m, 2H), 7.70 (d, *J* = 4.8 Hz, 2H), 7.64 (d, *J* = 8.3 Hz, 1H), 7.50 (dd, *J* = 12.0, 6.4 Hz, 4H), 7.43 (t, *J* = 7.8 Hz, 1H), 6.98 (d, *J* = 15.8 Hz, 1H), 5.72 (s, 2H). 13C NMR (100 MHz, DMSO-d_6_) *δ* 165.93, 152.85, 145.76, 145.76, 144.62, 137.60, 134.41, 132.52, 131.91, 131.81, 131.35, 129.66, 129.66, 128.79, 128.79, 128.20, 122.70, 120.64, 115.83, 115.83, 61.14.

##### 1-(4-Bromobenzyl)-4-cinnamamidopyridin-1-ium bromide (5g)

Yield 90%; yellow solid; IR (KBr) *ν* 3020, 1707, 1624, 1514, 1460, 1336, 1141, 972, 837, 762 cm^−1^; m.p. >250 °C; ESI/MS *m/z*: 393.0, 395.0 [M]^+^; ^1^H NMR (400 MHz, DMSO-d_6_) *δ* 11.81 (s, 1H), 8.92 (d, *J* = 7.4 Hz, 2H), 8.22 (d, *J* = 7.2 Hz, 2H), 7.79 (d, *J* = 15.8 Hz, 1H), 7.72–7.69 (m, 2H), 7.68–7.65 (m, 2H), 7.50–7.45 (m, 5H), 6.97 (d, *J* = 15.8 Hz, 1H), 5.70 (s, 2H). 13C NMR (100 MHz, DMSO-d_6_) *δ* 165.94, 152.81, 145.73, 145.73, 144.54, 134.47, 134.43, 132.56, 132.56, 131.35, 131.35, 129.65, 129.65, 128.77, 128.77, 123.06, 120.69, 115.73, 115.73, 61.19.

##### (E)-1-benzyl-4-(3-(3,4-dimethoxyphenyl) acrylamido) pyridin-1-ium bromide (5h)

Yield 91%; yellow solid; IR (KBr) *ν* 3087, 3022, 2962, 1706, 1641, 1596, 1512, 1463, 1264, 1132, 1025, 965, 746, 703 cm^−1^; m.p. >250 °C; ESI/MS *m/z*: 375.1 [M]^+^; ^1^H NMR (400 MHz, DMSO-d_6_) *δ* 11.74 (s, 1H), 8.95 (d, *J* = 7.4 Hz, 2H), 8.22 (d, *J* = 7.3 Hz, 2H), 7.73 (d, *J* = 15.6 Hz, 1H), 7.51–7.41 (m, 5H), 7.28 (dd, *J* = 6.4, 1.9 Hz, 2H), 7.06 (d, *J* = 8.9 Hz, 1H), 6.89 (d, *J* = 15.7 Hz, 1H), 5.72 (s, 2H), 3.85 (s, 3H), 3.80 (s, 3H). 13C NMR (100 MHz, DMSO-d_6_) *δ* 170.92, 157.60, 156.56, 154.20, 150.40, 150.40, 149.54, 139.97, 134.41, 134.41, 134.33, 133.72, 133.72, 131.95, 128.10, 122.89, 120.34, 117.04, 115.64, 115.64, 66.73, 60.90, 60.74.

##### (E)-4-(3-(3,4-dimethoxyphenyl) acrylamido)-1-(3-methylbenzyl) pyridin-1-ium bromide (5i)

Yield 93%; yellow solid; IR (KBr) *ν* 3016, 1697, 1626, 1509, 1458, 1257, 1134, 1015, 971, 840, 771, 709, 593 cm^−1^; m.p. >250 °C; ESI/MS *m/z*: 389.1 [M]^+^; ^1^H NMR (400 MHz, DMSO-d_6_) *δ* 11.69 (s, 1H), 8.91 (d, *J* = 7.4 Hz, 2H), 8.20 (d, *J* = 7.1 Hz, 2H), 7.73 (d, *J* = 15.6 Hz, 1H), 7.36–7.22 (m, 6H), 7.06 (d, *J* = 8.9 Hz, 1H), 6.84 (d, *J* = 15.7 Hz, 1H), 5.66 (s, 2H), 3.85 (s, 3H), 3.80 (s, 3H), 2.32 (s, 3H). 13C NMR (100 MHz, DMSO-d_6_) *δ* 166.17, 152.81, 151.81, 149.44, 145.59, 145.59, 144.83, 139.03, 135.07, 130.22, 129.57, 129.49, 127.18, 126.05, 123.39, 118.09, 115.59, 115.59, 112.27, 110.86, 62.04, 56.14, 55.99, 21.38.

##### (E)-4-(3-(3,4-dimethoxyphenyl) acrylamido)-1-(4-methylbenzyl) pyridin-1-ium bromide (5j)

Yield 94%; yellow solid; IR (KBr) *ν* 3068, 3014, 2948, 1683, 1622, 1508, 1462, 1293, 1132, 975, 794, 594 cm^−1^; m.p. >250 °C; ESI/MS *m/z*: 389.1 [M]^+^; ^1^H NMR (400 MHz, DMSO-d_6_) *δ* 11.40 (s, 1H), 8.65 (d, *J* = 6.7 Hz, 2H), 8.52 (s, 2H), 7.74 (d, *J* = 15.5 Hz, 1H), 7.48 (d, *J* = 15.5 Hz, 1H), 7.31–7.27 (m, 3H), 7.22–7.17 (m, 4H), 6.83 (d, *J* = 8.3 Hz, 1H), 5.65 (s, 2H), 3.95 (s, 3H), 3.90 (s, 3H), 2.35 (s, 3H). 13C NMR (100 MHz, DMSO-d_6_) *δ* 166.15, 152.77, 151.81, 149.45, 145.51, 145.51, 144.82, 139.17, 132.18, 130.18, 130.18, 129.07, 129.07, 127.02, 123.39, 118.10, 115.57, 115.57, 112.29, 110.85, 61.84, 56.16, 55.99, 21.22.

##### (E)-4-(3-(3,4-dimethoxyphenyl) acrylamido)-1-(3-fluorobenzyl) pyridin-1-ium bromide (5k)

Yield 88%; yellow solid; IR (KBr) *ν* 3014, 2964, 1711, 1688, 1592, 1518, 1445, 1258, 1137, 1014, 969, 850, 752, 571 cm^−1^; m.p. >250 °C; ESI/MS *m/z*: 393.1 [M]^+^; ^1^H NMR (400 MHz, DMSO-d_6_) *δ* 11.74 (s, 1H), 8.95 (d, *J* = 7.4 Hz, 2H), 8.22 (d, *J* = 7.0 Hz, 2H), 7.74 (d, *J* = 15.6 Hz, 1H), 7.55–7.47 (m, 1H), 7.42 (d, *J* = 9.8 Hz, 1H), 7.34 (d, *J* = 7.7 Hz, 1H), 7.27 (dd, *J* = 14.2, 4.5 Hz, 3H), 7.06 (d, *J* = 8.9 Hz, 1H), 6.88 (d, *J* = 15.7 Hz, 1H), 5.74 (s, 2H), 3.83 (d, *J* = 4.1 Hz, 6H). 13C NMR (100 MHz, DMSO-d_6_) *δ* 173.89, 165.13 (d, 1*J*_CF_=228.26 Hz), 161.48, 152.96, 151.82, 149.45, 145.71, 145.71, 144.85, 137.67 (d, 3*J*_CF_=8.16 Hz), 131.79 (d, 3*J*_CF_=8.27 Hz), 127.19, 125.19, 123.38, 118.10, 116.50 (d, 2*J*_CF_=21.52 Hz), 116.06 (d, 2*J*_CF_=22.38 Hz), 115.85, 115.62, 115.62, 112.28, 110.88, 61.19, 56.14, 55.98.

##### (E)-4-(3-(3,4-dimethoxyphenyl) acrylamido)-1-(4-fluorobenzyl) pyridin-1-ium bromide (5l)

Yield 91%; yellow solid; IR (KBr) *ν* 3080, 3015, 1703, 1621, 1597, 1506, 1461, 1263, 1131, 1021, 964, 844, 802, 786, 596 cm^−1^; m.p. >250 °C; ESI/MS *m/z*: 393.1 [M]^+^; ^1^H NMR (400 MHz, DMSO-d_6_) *δ* 11.69 (s, 1H), 8.91 (d, *J* = 7.3 Hz, 2H), 8.19 (d, *J* = 7.0 Hz, 2H), 7.72 (d, *J* = 15.6 Hz, 1H), 7.58 (dd, *J* = 8.7, 5.4 Hz, 2H), 7.31 (d, *J* = 8.9 Hz, 1H), 7.28 (dd, *J* = 4.2, 2.5 Hz, 2H), 7.05 (d, *J* = 8.9 Hz, 1H), 6.83 (d, *J* = 15.6 Hz, 1H), 5.69 (s, 2H), 3.82 (d, *J* = 3.7 Hz, 6H). 13C NMR (100 MHz, DMSO-d_6_) *δ* 166.17, 162.94 (d, 1*J*_CF_=251.49 Hz), 152.86, 151.82, 149.45, 145.55, 145.55, 144.82, 131.60 (d, 3*J*_CF_=8.08 Hz), 131.41, 127.19, 123.37, 118.11, 116.51 (d, 2*J*_CF_=21.47 Hz), 115.60, 112.28, 110.87, 61.13, 56.15, 55.98.

##### (E)-1-(3-bromobenzyl)-4-(3-(3, 4-dimethoxyphenyl) acrylamido) pyridin-1-ium bromide (5m)

Yield 92%; yellow solid; IR (KBr) *ν* 2928, 2831, 1592, 1511, 1460, 1313 1137, 990, 859, 762, 710 cm^−1^; m.p. >250 °C; ESI/MS *m/z*: 453.0, 455.0 [M]^+^; ^1^H NMR (400 MHz, DMSO-d_6_) *δ* 11.72 (s, 1H), 8.93 (d, *J* = 6.9 Hz, 2H), 8.21 (d, *J* = 6.5 Hz, 2H), 7.80 (s, 1H), 7.74 (d, *J* = 15.6 Hz, 1H), 7.64 (d, *J* = 8.0 Hz, 1H), 7.51 (d, *J* = 7.8 Hz, 1H), 7.42 (t, *J* = 7.9 Hz, 1H), 7.29 (s, 2H), 7.06 (d, *J* = 8.5 Hz, 1H), 6.85 (d, *J* = 15.6 Hz, 1H), 5.70 (s, 2H), 3.83 (d, *J* = 3.8 Hz, 6H). 13C NMR (100 MHz, DMSO-d_6_) *δ* 166.17, 152.96, 151.83, 149.45, 145.68, 144.90, 137.63, 132.51, 131.90, 131.81, 128.18, 127.17, 123.41, 122.70, 118.06, 115.68, 112.28, 110.87, 61.09, 56.15, 55.98.

##### (E)-1-(4-bromobenzyl)-4-(3-(3,4-dimethoxyphenyl) acrylamido) pyridin-1-ium bromide (5n)

Yield 88%; yellow solid; IR (KBr) *ν* 3012, 2948, 1681, 1618, 1508, 1460, 1268, 1130, 975, 841, 797 cm^−1^; m.p. >250 °C; ESI/MS *m/z*: 453.0, 455.0 [M]^+^; ^1^H NMR (400 MHz, DMSO-d_6_) *δ* 11.71 (s, 1H), 8.91 (d, *J* = 7.3 Hz, 2H), 8.20 (d, *J* = 6.9 Hz, 2H), 7.73 (d, *J* = 15.6 Hz, 1H), 7.67 (d, *J* = 8.4 Hz, 2H), 7.46 (d, *J* = 8.4 Hz, 2H), 7.32–7.25 (m, 2H), 7.06 (d, *J* = 8.9 Hz, 1H), 6.85 (d, *J* = 15.7 Hz, 1H), 5.69 (s, 2H), 3.82 (d, *J* = 3.7 Hz, 6H). 13C NMR (100 MHz, DMSO-d_6_) *δ* 166.17, 152.91, 151.83, 149.45, 145.66, 145.66, 144.87, 134.51, 132.57, 132.57, 131.32, 131.32, 127.18, 123.40, 123.05, 118.08, 115.62, 115.62, 112.28, 110.86, 61.18, 56.14, 55.98.

### Biological activity

#### *In vitro* inhibition studies on AChE and BuChE

Acetylcholinesterase (E.C. 3.1.1.7) from electric eel and human erythrocytes, butyrylcholinesterase (BuChE, E.C. 3.1.1.8) from equine serum and human serum, 5, 5′-dithiobis-(2-nitrobenzoic acid) (Ellman’s reagent, DTNB), *S*-butyrylthiocholine iodide (BTCI), acetylthiocholine iodide (ATCI) and donepezil hydrochloride were purchased from Sigma-Aldrich (St. Louis, MO). The capacity of the test compounds **5a**–**n** to inhibit AChE and BuChE activities was assessed by Ellman’s method^41^. Test compound was dissolved in a minimum volume of DMSO (1%) and was diluted using the buffer solution (50 mM Tris–HCl, pH =8.0, 0.1 M NaCl, 0.02 M MgCl_2_·6H_2_O). In 96-well plates, 160 μL of 1.5 mM DTNB, 50 μL of AChE (0.22 U/mL prepared in 50 mM Tris–HCl, pH =8.0, 0.1% w/v bovine serum albumin, BSA) or 50 μL of BuChE (0.12 U/mL prepared in 50 mM Tris–HCl, pH =8.0, 0.1% w/v BSA) were incubated with 10 μL of various concentrations of test compounds (0.001–100 μM) at 37 °C for 6 min followed by the addition of the substrate (30 μL) ATCI (15 mM) or BTCI (15 mM) and the absorbance was measured at different time intervals (0, 60, 120 and 180 s) at a wavelength of 405 nm. The concentration of compound producing 50% of enzyme activity inhibition (IC_50_) was calculated by nonlinear regression analysis of the response-concentration (log) curve, using the Graph-Pad Prism program package (Graph Pad Software, San Diego, CA). Results are expressed as the mean ± SEM of at least three different experiments performed in triplicate.

#### Kinetic analysis of AChE inhibition

To obtain the mechanism of action **5l**, reciprocal plots of 1/velocity versus 1/substrate were constructed at different concentrations of the substrate thiocholine iodide (0.05–0.5 mM) by using Ellman’s method^35^. Three concentrations of **5l** were selected for the studies: 30.0, 15.0 and 7.5 nM for the kinetic analysis of AChE inhibition. The plots were assessed by a weighted least-squares analysis that assumed the variance of velocity (*v*) to be a constant percentage of *v* for the entire data set. Slopes of these reciprocal plots were then plotted against the concentration of **5l** in a weighted analysis and Ki was determined as the intercept on the negative *x*-axis. Data analysis was performed with Graph Pad Prism 4.03 software (Graph Pad Software Inc., San Diego, CA).

#### Molecular modeling studies

Molecular modeling calculations and docking studies were performed using Molecular Operating Environment (MOE) software version 2008.10 (Chemical Computing Group, Montreal, Canada)^43^. The X-ray crystallographic structures of AChE (PDB code 1EVE) and A*β* (1–42) (PDB code PDB 1IYT) were obtained from the Protein Data Bank. All water molecules in PDB files were removed and hydrogen atoms were subsequently added to the protein. The compound **5l** was built using the builder interface of the MOE program and energy minimized using MMFF94x force field. Then the **5l** was docked into the active site of the protein by the “Triangle Matcher” method, which generated poses by aligning the ligand triplet of atoms with the triplet of alpha spheres in cavities of tight atomic packing. The Dock scoring in MOE software was done using ASE scoring function and force field was selected as the refinement method. The best 10 poses of molecules were retained and scored. After docking, the geometry of resulting complex was studied using the MOE’s pose viewer utility.

#### ABTS radical cation scavenging activity assay49

2,2′-Azino-bis-2-ethybenz-thiazoline-6-sulfonic acid (ABTS) was dissolved in purified water to a 7 mM concentration. ABTS radical cation (ABTS.+) was produced by reacting ABTS stock solution with 2.45 mM potassium persulfate (final concentration) and allowing the mixture to stand in the dark at room temperature for at least 18 h before use. The stock solution of ABTS was serially diluted with sodium phosphate buffer (50 mM, pH 7.4) to 100 μM. Trolox and **5a–n** at different concentrations (total volume of 50 μL) were added to 150 μL of 100 μM ABTS solution, respectively. After the addition of either trolox or another antioxidant to the ABTS solution, complete mixing of reactants was achieved by bubbling three to four times using plastic pipettes. The optical absorbance of ABTS at 415 nm was measured at 6 min after addition and equilibrated at 30 °C. Each individual treatment was repeated for three times and the results of the experiments were compared.

#### Inhibition of A*β* (1–42) self-induced aggregation

Inhibition of self-induced A*β* (1–42) aggregation was measured using a Thioflavin T (ThT)-binding assay^47^. HFIP pretreated A*β* (1–42) samples (Anaspec Inc., Fremont, CA) were resolubilized with a 50 mM phosphate buffer (pH 7.4) to give a 25 μM solution. Each tested compound was firstly prepared in dimethyl sulfoxide (DMSO) at a concentration of 10 mM and 1 μL of each was added to the well of black, opaque Corning 96-well plates such that the final solvent concentration was 10%. The final concentration of each compound was 20 μM and was prepared in independent triplicates. The solvent control was also included. Then, 9 μL of 25 mM A*β* (1–42) sample was added to each well and the samples mixed by gentle trapping. Plates were covered to minimize evaporation and incubated in dark at room temperature for 46–48 h with no agitation.

After the incubation period, 200 μL of 5 μM ThT in 50 mM glycine–NaOH buffer (pH 8.0) was added to each well. Fluorescence was measured on a SpectraMax M5 (Molecular Devices, Sunnyvale, CA) multi-mode plate reader with excitation and emission wavelengths at 446 nm and 490 nm, respectively. The fluorescence intensities were compared and the percent inhibition due to the presence of the inhibitor was calculated by the following formula: 100 − (IF_i_/IF_o_×100) where IF_i_ and IF_o_ are the fluorescence intensities obtained for A*β* (1–42) in the presence and in the absence of inhibitor, respectively.

#### Metal-chelating study

The study of metal chelation was performed in methanol at 298 K using UV–vis spectrophotometer (SHIMADZU UV-2450PC, Kyoto, Japan) with wavelength ranging from 200 to 500 nm. Due to the low solubility in PBS, the compounds were tested in methanol. The absorption spectra of compound **5l** (50 μM, final concentration) alone or in the presence of CuSO_4_ and FeSO_4_ (100 μM, final concentration) for 30 min in methanol were recorded 1 cm-quartz cells. A fixed amount of compound **5l** (50 μM) was mixed with growing amounts of Cu^2+^ (10–80 μM) and UV spectra were recorded. Variation of absorbance at 363 nm was used to monitor the formation of **5l**/Cu^2+^ complex^44^^,^^45^.

#### Inhibition of Cu^2+^-induced A*β* (1–42) aggregation

For the inhibition of Cu^2+^-induced A*β* (1–42) aggregation experiment, the A*β* was diluted in 20 μM HEPES (pH 6.6) with 150 μM NaCl. The mixture of the peptide (10 μL, 25 μM, final concentration) with or without copper (10 μL, 25 μM, final concentration) and the test compound (10 μL, 50 μM, final concentration) were incubated at 37 °C for 24 h. The 20 μL of the sample was diluted to a final volume of 200 μL with 50 μM glycine–NaOH buffer (pH 8.0) containing ThT (5 μM). The detection method was the same as that of self-induced A*β* aggregation experiment^47^.

#### Cell culture and measurement of cell viability^50^

The toxicity effect of the tested compounds on the rat pheochromocytoma (PC12) cells was examined according to previously reported methods. PC 12 cells was obtained from the Cell Bank of the Chinese Academy of Sciences (Shanghai, China) and routinely grown at 37 °C in a humidified incubator with 5% CO_2_ in Dulbecco’s modified Eagle’s medium (DMEM) supplemented with 10% bovine calf serum, 100 units per mL penicillin, and 100 units per mL of streptomycin. Cells were sub-cultured in 96-well plates at a seeding density of 5000 cells per well and allowed to adhere and grow. When cells reached the required confluence, they were placed into serum-free medium and treated with compound **5l**. Twenty-four hours later the survival of cells was determined by MTT assay. Briefly, after incubation with 20 μL of MTT at 37 °C for 4 h, living cells containing MTT formazan crystals were solubilized in 200 μL DMSO. The absorbance of each well was measured using a microculture plate reader with a test wavelength of 570 nm and a reference wavelength of 630 nm. Results are expressed as the mean ± SD of three independent experiments.

PC 12 cells were grown in RPMI-1640 medium containing 10% (v/v) foetal bovine serum, 100 U penicillin/mL and 100 mg streptomycin/mL under 5% CO_2_ at 37 °C. The culture media were changed every other day. After pretreatment with different concentrations of test compound (0, 6, 12.5, 25, 50 μM) for 2 h, PC 12 cells were incubated with 25 μM of A*β* (1–42) for 24 h. The cell viability was evaluated using MTT assay. Briefly, the cells were treated with 10 μL MTT (5 mg/mL in PBS) for 4 h at 37 °C. Then, 200 μL of DMSO was added to dissolve the dark blue formazan crystals formed in intact cells, and the absorbance at 570 nm was detected by a microplate reader. PC12 cells were cultured without test compound or A*β* (1–42) as control group and the results were expressed by percentage of control.

#### *In vitro* BBB permeation assay

Brain penetration of compounds was evaluated using a parallel artificial membrane permeation assay (PAMPA) in a similar manner as described by Di et al.^51^ Commercial drugs were purchased from Sigma (St. Louis, MO) and Alfa Aesar (Haverhill, MA). The porcine brain lipid (PBL) was obtained from Avanti Polar Lipids (Alabaster, AL). The donor microplate (PVDF membrane, pore size 0.45 mm) and the acceptor microplate were both from Millipore (Darmstadt, Germany). The 96-well UV plate (COSTAR*^@^*) was from Corning Incorporated (Harrodsburg, KY). The acceptor 96-well microplate was filled with 300 μL of PBS/EtOH (7:3), and the filter membrane was impregnated with 4 μL of PBL in dodecane (20 mg/mL). Compounds were dissolved in DMSO at 5 mg/mL and diluted 50-fold in PBS/EtOH (7:3) to achieve a concentration of 100 mg/mL, 200 μL of which was added to the donor wells. The acceptor filter plate was carefully placed on the donor plate to form a sandwich, which was left undisturbed for 16 h at 25 °C. After incubation, the donor plate was carefully removed and the concentration of compound in the acceptor wells was determined using an UV plate reader (Flexstation*^@^* 3). Every sample was analyzed at five wavelengths, in four wells, in at least three independent runs, and the results are given as the mean ± SD. In each experiment, nine quality control standards of known BBB permeability were included to validate the analysis set.

#### *Ex vivo* brain penetration^52^

Fifteen male OF1 mice, weighing 25 g were used. Animals were housed under controlled light (with a 12-h light/12-h dark cycle, lights on at 7:00 a.m.) at 25 °C and proper humidity. Rats were given food and tap water *ad libitum*.

Donepezil hydrochloride and compound **5l** were dissolved 10% DMSO. Animals were divided into three experimental handling groups: mice administered with (i) control (10% DMSO, *n* = 5); (ii) donepezil (10 μmol/kg, *n* = 5); (iii) compound **5l** (10 μmol/kg, *n* = 5). Groups of 15 mice were treated i.p. with each compound. The animals were sacrificed 10 min later and brains were quickly removed and frozen on dry ice. Residual AChE activity was determined as previously described by the method of Ellman et al.^41^ using brain homogenate preparations as a source of the enzyme: A homogenate of brain samples (10%, w/v) in 0.03 M sodium phosphate buffer (pH 7.4) was prepared. The brain homogenate in volume of 200 μL was mixed with 1% Triton X-100 and centrifuged at 3000 rpm at 4 °C for 10 min. Just before analysis of the enzymatic activity, an amount of 100 μL of homogenate was diluted 2.5 times in 0.1 M phosphate-buffered solution (pH 8.0). The reaction took place in a final volume of 300 μL of 0.1 M phosphate-buffered solution (pH 8.0) containing 100 μL of diluted homogenate and 333 μM DTNB solution. To avoid interferences between AChE and BChE activities, 100 μM ISOOMPA (specific BChE inhibitor) was present in the incubation medium. Prior to the addition of the substrate acetylthiocholine, a preincubation period of 5 min was used to eliminate the endogenous ACh present in the homogenates. The reaction was started by the addition of ATCI (450 μM) and the absorbance at 414 nm was evaluated 2 min after the substrate addition. Percent of inhibition was calculated by comparing AChE activity in brain of the drug-treated mice with activity from untreated controls.

## Result and discussion

### Chemistry

**Scheme 1.**
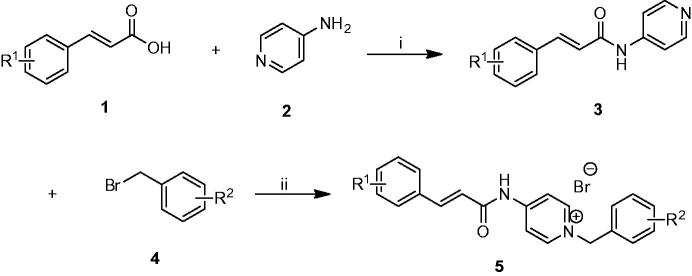
Synthesis of cinnamic acid derivatives **5a–n**. Reagents and conditions: (i) DMAP/EDCI, CH_2_Cl_2_, rt., 12 h; (ii) CH3CN, reflux, 1–3 h.

As shown in Scheme 1, the target compounds **5a**–**n** were synthesized. First of all, the commercially available cinnamic acid derivatives **1** were activated with 4-dimethylaminopyridine (DMAP) and 1-(3-dimethylaminopropyl)-3-ethylcarbodiimide hydrochloride (EDCI) at 0 °C in dichloromethane solutions, and subsequently condensation with compound **2** at room temperature overnight afforded the intermediates **3**. Finally, the benzyl pyridinium bromide salts **5** were efficiently obtained by refluxing proper benzyl bromides **4** with the intermediates **3** in dry acetonitrile.

### *In vitro* cholinesterase inhibitory activity and preliminary SAR studies

The activities of compounds **5a**–**n** and the relative compound cinnamic acid against AChE (from electric eel) and BuChE (from equine serum) were examined using the spectrophotometric method of Ellman et al.^41^ Donepezil was used as a standard compound for comparison. From Table 1, the novel cinnamic acid derivatives showed high activity towards AChE with IC_50_ values in the nanomolar range, and high selectivity for AChE over BuChE, indicating that these derivatives are selective AChE inhibitors. Among the cinnamic acid derivatives, compound **5l** (IC_50_=12.1 nM) showed the most potent inhibitory activity against AChE, which was 3.3-fold higher than that of donepezil (IC_50_=40.2 nM). Moreover, compound **5l** exhibited the highest selectivity level towards AChE over BuChE (SI =214.9). By contrast, compound **5n** exhibited the highest inhibitory activity against BuChE (IC_50_=1.9 μM), resulting 2.3-times more potent than that of donepezil (IC_50_=4.5 μM). However, the AChE inhibitory activity of cinnamic acid was remarkably low (IC_50_>100 μM), suggesting that the *N*-benzyl pyridinium moiety is unavoidably required for higher activity. The AChE inhibition by benzyl pyridinium bromide was assayed to be in the micromolar range^40^, which was lower than that of novel cinnamic acid derivatives. This finding suggests that the cinnamic acid skeleton was also essential for AChE activity.

**Table 1.** Inhibition of ChEs activity and selectivity index of compounds **5a–n**.

			IC_50_^a^		
Compound	R^1^	R^2^	*ee*AChE (nM)	*e*qBuChE (μM)	Selectivity index (SI)^b^
**5a**	–	–	54.1 ± 8.2	6.3 ± 0.6	116.5
**5b**	–	3-CH_3_	102.6 ± 9.6	8.1 ± 1.3	78.9
**5c**	–	4-CH_3_	1263.8 ± 1.2	25.0 ± 0.7	19.8
**5d**	–	3-F	93.5 ± 7.7	7.4 ± 0.5	79.1
**5e**	–	4-F	90.8 ± 9.2	10.0 ± 0.4	110.1
**5f**	–	3-Br	153.6 ± 2.6	2.5 ± 0.3	16.3
**5g**	–	4-Br	1450.5 ± 4.3	2.1 ± 0.8	1.5
**5h**	3,4-di-OCH_3_	–	135.2 ± 4.8	4.5 ± 0.7	33.3
**5i**	3,4-di-OCH_3_	3-CH_3_	51.4 ± 18	2.7 ± 0.8	52.3
**5j**	3,4-di-OCH_3_	4-CH_3_	90.7 ± 3.2	3.8 ± 0.9	41.9
**5k**	3,4-di-OCH_3_	3-F	20.6 ± 1.0	3.1 ± 0.5	150.5
**5l**	3,4-di-OCH_3_	4-F	12.1 ± 1.8	2.6 ± 1.6	214.9
**5m**	3,4-di-OCH_3_	3-Br	92.6 ± 9.0	2.5 ± 0.6	27.0
**5n**	3,4-di-OCH_3_	4-Br	50.3 ± 8.3	1.9 ± 0.5	37.8
**Cinnamic acid**	–	–	>100 000	>100	–
**Donepezil**	–	–	40.2 ± 3.6	4.5 ± 0.2	112.5

a IC_50_: 50% inhibitory concentration (means ± SD of three experiments).

b Selectivity Index = IC_50_ (*e*qBuChE)/IC_50_ (*ee*AChE).

To improve the inhibitory activity of the compounds against ChEs, we introduced substituents with different sizes and electronic properties on the benzene ring of cinnamic acid and on the benzyl group of the *N*-benzyl pyridinium moiety were varied. The IC_50_ value of compounds **5a**–**n** indicated that the presence of methoxy groups at the positions 3 and 4 of the *N*-benzyl pyridinium moiety increased the ChE activity. For example, the ChE inhibitory activity of compounds **5i–n** was higher than that of compounds **5b**–**g**. Moreover, AChE inhibition was also affected by the substituents on the benzyl group. Compared with compound **5a**, the introduction of methyl, fluorine and bromine on the meta-, para-position reduced AChE inhibitory activity (compounds **5b**–**g**). For example, compound **5a** (IC_50_=54.1 nM for AChE) was more potent than compound **5g** (IC_50_=1450.5 nM for AChE) possessing 4-Br on the benzyl group. By contrast, compared with compound **5h**, compounds **5i**–**n** with different substituents on the benzyl group showed an increased AChE inhibitory activity. For example, compound **5l** bearing 4-F on the benzyl group exhibited more potent AChE inhibition (11-fold) than did compound **5h**. Similar to compound **5a**–**n** for BuChE inhibition, compounds **5f** (IC_50_=2.5 μM), **5g** (IC_50_=2.1 μM), **5l** (IC_50_=2.5 μM) and **5m** (IC_50_=1.9 μM) that were characterized by a Br substituent showed more potent inhibition. Therefore, we can rationally deduce that the size of a substituent more crucially affects BuChE inhibition than its electronic properties.

Finally compounds **5i**–**n** were selected for evaluation on human AChE. As listed in Table 2, all tested compounds presented IC_50_ values in the nanomolar range and were slightly more potent inhibitors for *h*AChE than for *ee*AChE. The SARs for *h*AChE were similar to those drawn for *ee*AChE inhibition (Table 1). Compound **5l** (IC_50_=8.6 nM for *h*AChE) displayed the highest inhibition, which was fourfold higher than that of standard donepezil (IC_50_=33.5 nM).

**Table 2.** Inhibition of ChEs activity and selectivity index of compounds **5i–n**.

	IC_50_^a^		
Compounds	*h*AChE (nM)	*h*BuChE (μM)	Selectivity index^b^
**5i**	43.7 ± 0.5	4.9 ± 0.7	112.1
**5j**	96.5 ± 2.8	5.6 ± 1.9	58.0
**5k**	19.2 ± 1.6	4.8 ± 0.5	250.0
**5l**	8.6 ± 0.9	4.4 ± 1.2	511.6
**5m**	79.4 ± 1.5	2.1 ± 0.6	26.5
**5n**	51.1 ± 4.8	1.9 ± 0.5	37.2
**Donepezil**	33.5 ± 3.8	7.6 ± 1.9	226.9

a IC_50_: 50% inhibitory concentration (means ± SD of three experiments).

b Selectivity Index = IC_50_ (*h*BuChE)/IC_50_ (*h*AChE).

### Kinetic study of AChE inhibition

**Figure 2.**
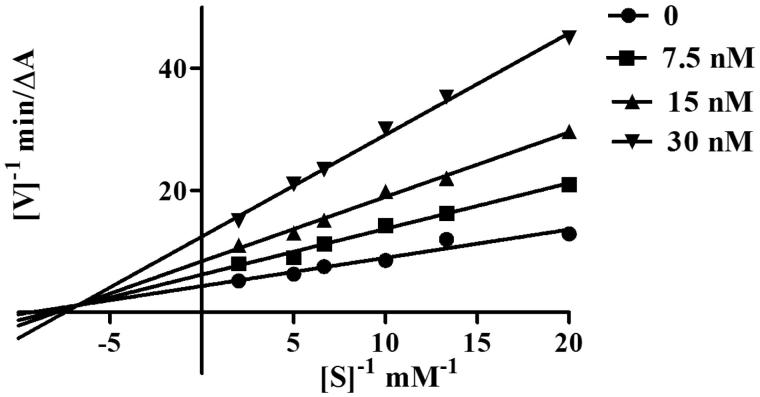
Kinetic study on the mechanism of *Ee*AChE inhibition by compound **5l**. Overlaid Lineweaver–Burk reciprocal plots of AChE initial velocity at increasing substrate concentration (0.05–0.50 mM) in the absence of inhibitor and in the presence of compound **5l** are shown. Lines were derived from a weighted least-squares analysis of the data points.

To explore the AChE inhibitory mechanism of action of the cinnamic acid derivatives, the most potent inhibitor, compound **5l**, was selected for a kinetic study using Lineweaver–Burk plots^39^. Graphical analysis (Figure 2) revealed both increasing slopes and increasing intercepts with increasing inhibitor concentration. According to this pattern, compound **5l** is a mixed-type inhibitor for AChE and might be able to bind to the CAS and the PAS of AChE.

### Molecular modeling study of AChE inhibition

**Figure 3.**
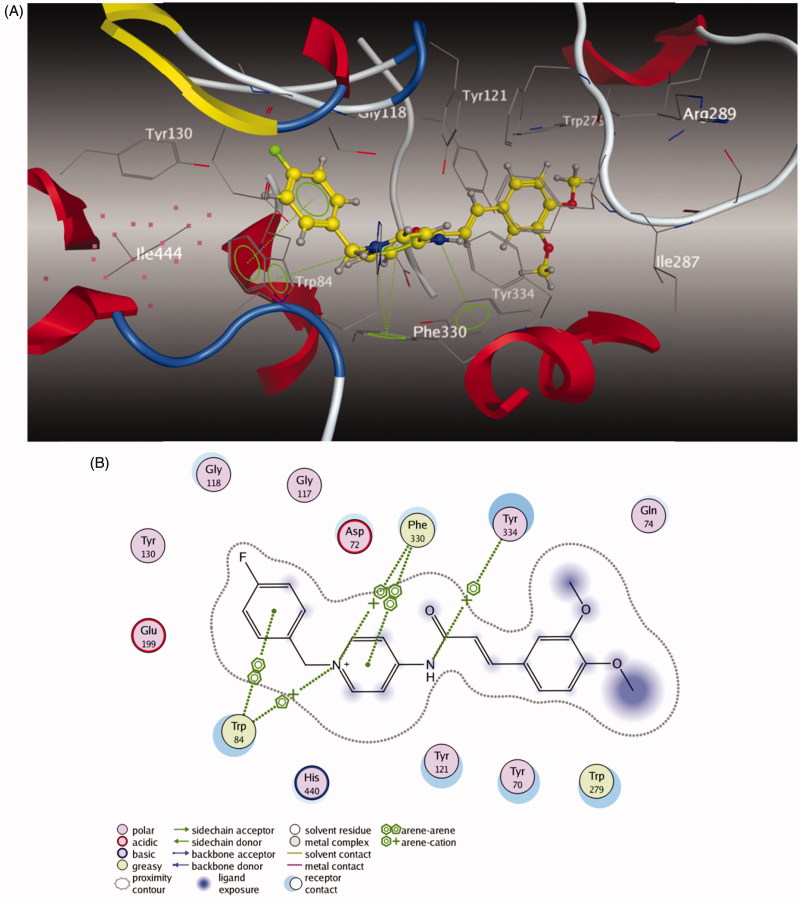
(A) 3D docking model of compound **5l** with *Tc*AChE. Atom colors: yellow – carbon atoms of **5l**, gray – carbon atoms of residues of *Tc*AChE, dark blue – nitrogen atoms, red – oxygen atoms. The dashed lines represent the interactions between the protein and the ligand. (B) 2D schematic diagram of docking model of compound **5l** with *Tc*AChE. The figure was prepared using the ligand interactions application in MOE.

To further study the dual-site mode of compound **5l** for AChE, a molecular docking study was performed using the software package MOE 2008.10^42,43^. The X-ray crystal structure of the *Tc*AChE complex with donepezil (*h*AChE, PDB code 1EVE) was applied to establish the starting model of AChE. As shown in Figure 3, the *N*-benzyl pyridinium moiety of compound **5l** was bound to the CAS of AChE, via aromatic *π*–*π* stacking interactions with the phenyl ring from Trp 84 with the ring-to-ring distance of 3.88 Å and the pyridine ring from Phe 330 with the ring-to-ring distance of 349 Å. Moreover, the charged nitrogen of the pyridine ring bound to the CAS was via a cation–*π* interaction with Trp 84 and Phe 330. The cinnamic acid moiety occupied the PAS formed by Trp 279 and Gln 74. All these results obviously indicated that compound **5l** could simultaneously bind to the PAS and CAS of AChE.

### Metal-chelating properties of compound 5l

**Figure 4.**
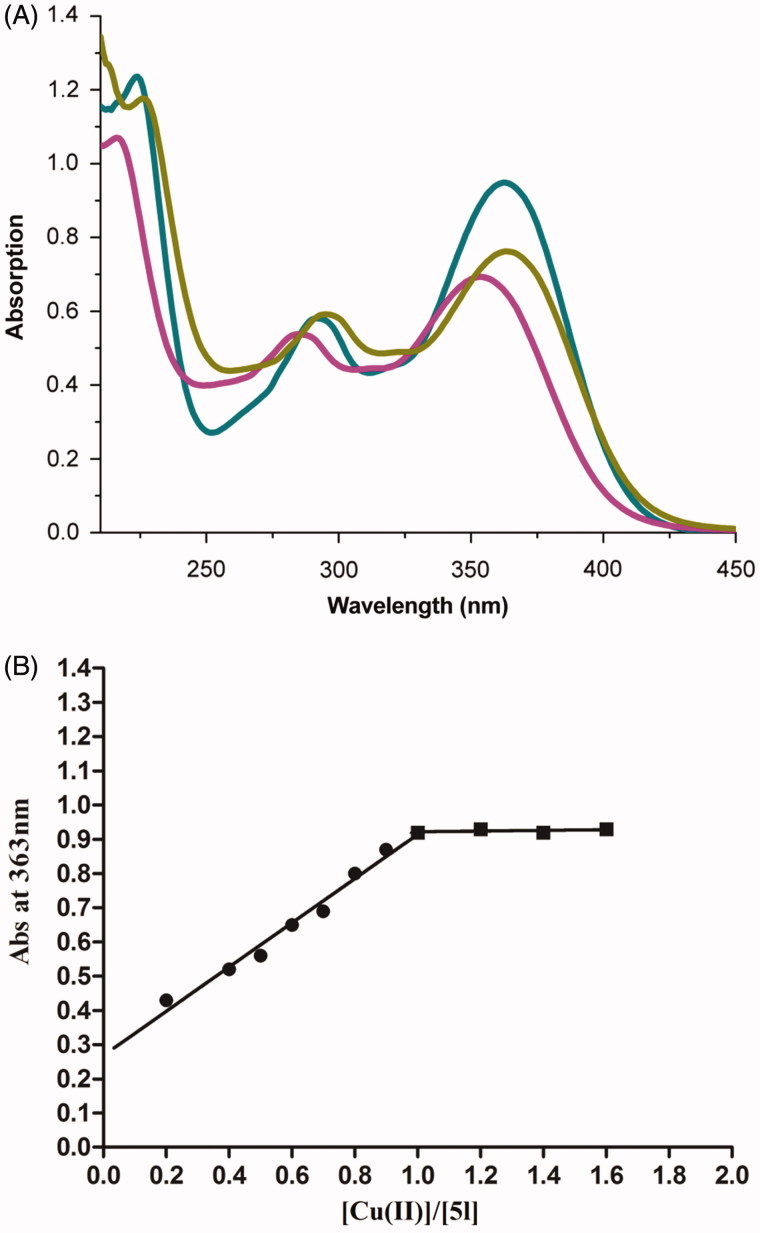
(A) UV absorbance spectrum of **5l** (50 μM) alone (red) or in the presence of 100 μM CuSO_4_ (green) and 100 μM FeSO_4_ (yellow) in MeOH. (B) Determination of the stoichiometry of complex **5l**−Cu^2+^ by molar ratio method.

The chelating ability of compound **5l** with biometals such as Cu^2+^ and Fe^2+^ was studied using UV–vis spectroscopy (Figure 4)^44^^,^^45^. As shown in the UV–vis spectrum (Figure 4(A)), compound **5l** without metal ions showed the absorption maximum at 216 nm and a shoulder at 353 nm. Upon the addition of CuSO_4_, a bathochromic shift in the maximum absorption from 216 nm to 224 nm and in the shoulder from 353 nm to 363 nm occurred, suggesting the formation of the **5l**–Cu^2+^ complex. When FeSO_4_ was added, a red shift in the maximum absorption from 216 nm to 226 nm occurred, suggesting that compound **5l** coordinates with Fe^2+^. The complexation ability of compound **5l** might be ascribed to the dimethoxy group on the cinnamic acid moiety and to the amide moiety of the compound^46^^,^^33^.

To determine the stoichiometry of the **5l–**Cu^2+^ complex, the molar ratio method was used, for which solutions of compound **5l** with accumulative amounts of CuSO_4_ were prepared. The UV spectra (Figure 4(B)) showed that the absorbance at 363 nm, related to the formation of the Cu–**5l** complex, initially increased at increasing concentrations of CuCl_2_ and then plateaued, and the two straight lines intersected at a mole fraction of 1.02. Thus a 1:1 stoichiometry was hypothesised for the **5l–**Cu^2+^ complex.

### Inhibition of self-induced and Cu^2+^-induced A*β* (1–42)self-induced aggregation

All compounds tested for ChEs inhibition were also evaluated by a ThT-based fluorometric assay for their ability to inhibit self-induced A*β* (1–42) aggregation^47^. Curcumin was used as a reference, because of its known inhibitory activity against A*β* (1–42) self-aggregation. The results are gathered in Table 3. Compounds **5a**–**n** exhibited good potencies (46.3–65.6% at 20 μM) compared with Cur (54.6% at 20 μM). Notably, compounds **5k**, **5l** and **5n** (65.6%, 64.7% and 66.3%, respectively, at 20 μM) showed the highest potency. Interestingly, compounds **5h**–**n** with the dimethoxy group on the cinnamic acid moiety exhibited high inhibition against self-induced A*β* (1–42) aggregation with inhibition ranging from 53.9 to 66.3% at 20 μM. For example, compound **5l** (64.7% at 20 μM) was more potent than that of compound **5e** (52.8% at 20 μM). This finding led to the hypothesis that the dimethoxy group might favour A*β* aggregation inhibition. However, substituents on the benzyl group (see compounds **5i–n**) did not seem to play a role in the inhibition of A*β* (1–42) self-aggregation.

**Table 3.** Inhibition of A*β* (1–42) self-induced aggregation and ABTS radical by target compounds.

Compound	R^1^	R^2^	A*β* (1–42) aggregationinhibition (%)^a^	ABTS assay(trolox equiv)^b^
**5a**	–	–	46.3 ± 1.6	N^c^
**5b**	–	3-CH_3_	48.5 ± 1.8	N
**5c**	–	4-CH_3_	55.1 ± 1.5	N
**5d**	–	3-F	53.6 ± 2.3	N
**5e**	–	4-F	52.8 ± 1.8	N
**5f**	–	3-Br	48.8 ± 2.1	N
**5g**	–	4-Br	57.2 ± 1.1	N
**5h**	3,4-di-OCH_3_	–	55.7 ± 3.2	N
**5i**	3,4-di-OCH_3_	3-CH_3_	53.9 ± 0.9	N
**5j**	3,4-di-OCH_3_	4-CH_3_	63.4 ± 2.8	N
**5k**	3,4-di-OCH_3_	3-F	65.6 ± 2.3	N
**5l**	3,4-di-OCH_3_	4-F	64.7 ± 1.6	N
**5m**	3,4-di-OCH_3_	3-Br	62.8 ± 1.4	N
**5n**	3,4-di-OCH_3_	4-Br	66.3 ± 2.9	N
**Curcumin**	–	–	54.6 ± 2.6	1.65

a Inhibition of A*β* (1–42) self-induced aggregation, the thioflavin-T fluorescence method was used, the mean ± SD of at least three independent experiments and the measurements were carried out in the presence of 20 μM compounds.

b Data are expressed as (mmol trolox)/(mmol tested compound).

c N means <0.05.

**Figure 5.**
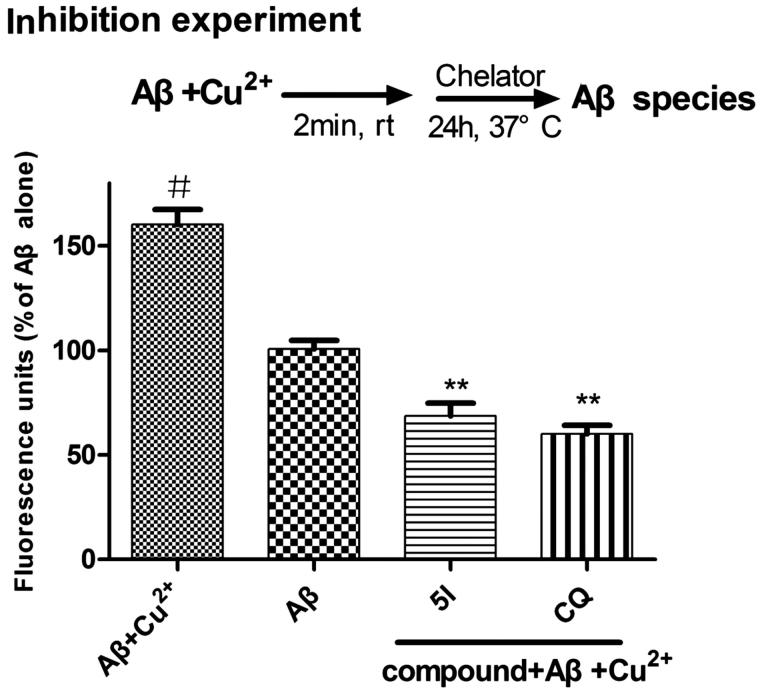
Inhibition of Cu^2+^-induced A*β* (1–42) aggregation by compound **5l** comparing with that of clioquinol (CQ) ([A*β* = 25 μM, [**5l**] = 50 μM, [CQ] = 50 μM, [Cu^2+^] = 25 μM, 37 °C, 24 h). Values are reported as the mean ± SD of three independent experiments. #*p* < .05, **p < .01.

As compound **5l** showed good inhibitory activity against A*β* (1–42) self-aggregation and favourable chelating properties, its ability to inhibit Cu^2+^-induced A*β* (1–42) aggregation was investigated by a ThT-binding assay^48^. Clioquinol was employed as a reference compound. As shown in Figure 5, the fluorescence of A*β* treated with Cu^2+^ is 160.3% that of A*β* alone, which points out that Cu^2+^ hastens A*β* aggregation. In comparison, the fluorescence of A*β* treated with Cu^2+^ and the tested compound decreased dramatically (**5l**, 68.6% inhibition of Cu^2+^-induced A*β* aggregation; CQ, 60.2% inhibition). These results indicated that our compound could inhibit Cu^2+^-induced A*β* aggregation by effectively chelating Cu^2+^.

### Docking study of compound 5l with A*β* (1–42) peptide

**Figure 6.**
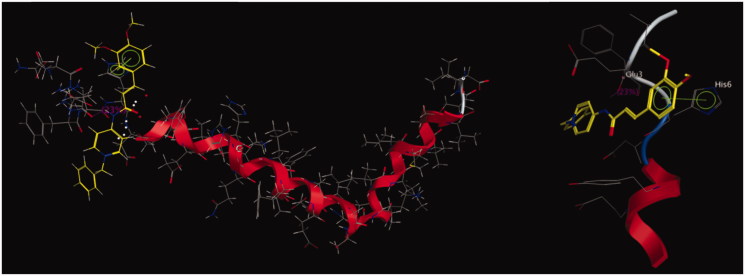
Docking study of compound **5l** with A*β* (1–42) generated with MOE. Atom colors: yellow – carbon atoms of **5l**, gray – carbon atoms of residues of A*β* (1–42), dark blue – nitrogen atoms, red – oxygen atoms. The dashed lines represent the interactions between the protein and the ligand.

To further study the interaction mode with compound **5l** for A*β* (1–42), a molecular docking study was performed using the software package MOE 2008.10^43^. The X-ray crystal structure of the protein A*β* structure (PDB 1IYT) from the Protein Data Bank was used. As revealed in Figure 6, the benzene ring of cinnamic acid interacted with the His 6 via a *π*–*π* stacking interaction. A hydrogen bond interaction was also observed between the acid amides group of compound **5l** and Glu 3. These results indicated that the *π*–*π* stacking and the hydrogen bond interactions played crucial roles in the stability of the **5l**/A*β* (1–42) complex.

### Evaluation of compounds for antioxidant activity

The target compounds were evaluated for their antioxidant efficacy by using ABTS (ferric reducing antioxidant power) assays^49^. Compared with Cur, results showed that tested compounds exhibited no antioxidant ability (Table 3). This finding might be attributed to the absence of the hydroxy group of cinnamic acid moiety.

### Cytotoxicity of compound 5l in PC12 cells and neuroprotection against A*β* (1–42)-induced toxicity

**Figure 7.**
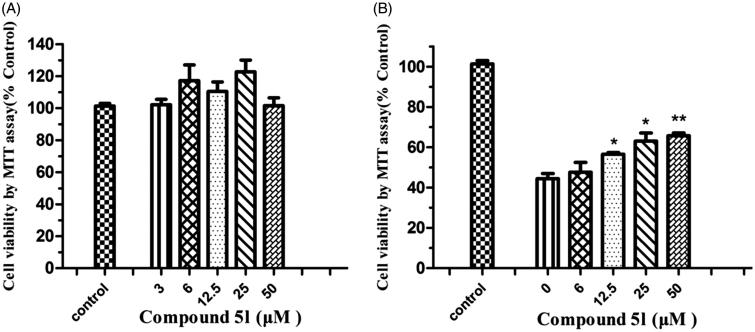
(A) Effects of compound **5l** on cell viability in PC12 cells. The cell viability was determined by the MTT assay after 24 h of incubation with various concentrations. The results were expressed as a percentage of control cells. Values are reported as the mean ± SD of three independent experiments. (B) Neuroprotection against A*β* (1–42) toxicity. Compound **5l** was tested for neuroprotective activity against A*β* (1–42) toxicity in PC12 cells. Data represent the mean SD of three observations. *p < .05 and **p < .01 compared to the A*β* (1–42)-treated control group.

On the basis of the aforementioned screening results, the potential toxicity effect of compound **5l** in PC12 cells was studied^50^. After incubating the cells with compound **5l** for 24 h, the cell viability was determined by the 3-(4,5-dimethylthiazol-2-yl)-2,5-diphenyltetrazolium (MTT) assay. As shown in Figure 7(A), the result revealed that compound **5l** at 3–50 μМ did not significantly affect cell viability, indicating that compound **5l** was nontoxic to neuroblastoma PC12 cells.

In the pathology of AD, A*β*-induced neuronal cell death is a serious event. To evaluate the neuroprotective effects of compound **5l** against A*β*-induced neuronal death of PC12 cells, the data were recorded after the cells were exposed to increasing concentrations of compound **5l** (6, 12.5, 25 and 50 μM) for 24 h. As can be seen in Figure 7(B), treatment of cells with A*β* (1–42) (25 μM) to the growth medium markedly reduced cell viability to 44.2% compared with the untreated cells (control). Compound **5l** exhibited neuroprotective effects at concentrations ranging from 6 to 50 μM (6 μM: 47.6 ± 4.2%; 12.5 μM: 57.5 ± 1.4%; 25 μM: 63.2 ± 3.5%; 50 μM: 66.6 ± 2.5%). These observations further showed that novel cinnamic acid derivatives bearing the *N*-benzyl pyridinium moiety can inhibit A*β* (1–42) self-aggregation for the treatment of AD.

### *In vitro* BBB permeation assay

For successful central nervous system (CNS) drugs, the first requirement is crossing the BBB to reach brain. The potential ability of these compounds to penetrate into the brain was evaluated using a PAMPA as described by Di et al.^51^ Assay validation was completed by comparing the experimental permeabilities of nine commercial drugs with previously reported values (Table 4)^51^. A plot of the experimental data versus bibliographic values gave a good linear correlation, *P*_e_ (exp.) = 1.06 *P*_e_ (bibl.) − 1.69 (*R*^2^=.94). From this equation and taking into account the limit established by Di et al. for blood–brain barrier permeation, we classified the compounds as follows:

**Table 4.** Permeability (*P*_e_×10^−6 ^cm s^−1^) in the PAMPA-BBB assay for nine commercial drugs, used in the experiment validation.

Commercial drugs	Bibl^a^	PBS:EtOH(70:30)^b^
Testosterone	17	16.44
Verapamil	16	16.88
beta-Estradiol	12	11.93
Progesterone	9.3	5.34
Corticosterone	5.1	4.06
Piroxicam	2.5	1.42
Hydrocortisone	1.9	1.86
Ofloxacin	0.8	0.47
Dopamine	0.2	0.17

a Taken from Ref.^49^

b Data are the mean ± SD of three independent experiments.

- “CNS +” (high BBB permeation predicted): *P*_e_ (10^−^6 cm s^−1^) > 2.55.
- “CNS −” (low BBB permeation predicted): *P*_e_ (10^−^6 cm s^−1^) < 0.43.
- “CNS +/−” (BBB permeation uncertain): *P*_e_ (10^−6^ cm s^−1^) from 2.55 to 0.43.

Compound **5l** with good activities against A*β* (1–42) aggregation and AChE was selected. The *P*_e_ value of compound **5l** was 2.89 ± 0.37 (CNS+). It indicated that compound **5l** might be able to penetrate the BBB.

### *Ex vivo* brain penetration study

To confirm the brain permeability of compound **5l** predicted by the PAMPA, the AChE inhibitory activity was subjected to an *ex vivo* measurement after intraperitoneal (i.p.) injection of 10 μmol/kg of compound **5l** and donepezil into mice^52^. The mice were sacrificed 10 min after drug administration, and the inhibition (%) of brain AChE activity versus untreated controls was determined. Compared to control, mouse brain AChE activity was found to be inhibited by compound **5l** (53.9 ± 2.9%) and donepezil (46.7 ± 3.2%). These results confirmed that compound **5l** can cross the BBB *in vivo*.

## Conclusions

In summary, novel cinnamic acid derivatives bearing *N*-benzyl pyridinium moiety were designed, synthesised and evaluated as multifunctional cholinesterase inhibitors against AD. Among the synthesised compounds, most derivatives displayed potent AChE inhibitory activity and high selectivity for AChE over BuChE. Among them, compound **5l** exhibited dual inhibitory potency on AChE and BuChE. The kinetic characterization suggested that compound **5l** acted as a mixed-type inhibition, which was consistent with the result of the molecular modelling study. Furthermore, compound **5l** showed metal-chelating ability, significant inhibition of A*β* aggregation and inhibition of Cu^2+^-induced A*β* aggregation, in addition to low neurotoxicity. Compound **5l** also showed a neuroprotective effect against A*β* (1–42) toxicity in PC12 cells and was proven to penetrate into brain by the PAMPA-BBB assay *in vitro* and *ex vivo* experiments. Above all, compound **5l** could be deemed as a multifunctional cholinesterase inhibitor and serve as a novel lead compound for treating AD.
